# Keep in touch (KIT): perspectives on introducing internet-based communication and information technologies in palliative care

**DOI:** 10.1186/s12904-016-0140-5

**Published:** 2016-08-02

**Authors:** Qiaohong Guo, Beverley Cann, Susan McClement, Genevieve Thompson, Harvey Max Chochinov

**Affiliations:** 1 Department of Psychiatry, University of Manitoba, Winnipeg, Canada; 2 Manitoba Palliative Care Research Unit, CancerCare Manitoba, 3017-675 McDermot Ave, Winnipeg, R3E 0V9 MB Canada; 3 College of Nursing, University of Manitoba, Winnipeg, Canada

**Keywords:** Communication and information technology, Palliative care, Palliative inpatients, Family members

## Abstract

**Background:**

Hospitalized palliative patients need to keep in touch with their loved ones. Regular social contact may be especially difficult for individuals on palliative care in-patient units due to the isolating nature of hospital settings. Technology can help mitigate isolation by facilitating social connection. This study aimed to explore the acceptability of introducing internet-based communication and information technologies for patients on a palliative care in-patient unit.

**Methods:**

In the first phase of the Keep in Touch (KIT) project, a diverse group of key informants were consulted regarding their perspectives on web-based communication on in-patient palliative care units. Participants included palliative patients, family members, direct care providers, communication and information technology experts, and institutional administrators. Data was collected through focus groups, interviews and drop-in consultations, and was analyzed for themes, consensus, and major differences across participant groups.

**Results:**

Hospitalized palliative patients and their family members described the challenges of keeping in touch with family and friends. Participants identified numerous examples of ways that communication and information technologies could benefit patients’ quality of life and care. Patients and family members saw few drawbacks associated with the use of such technology. While generally supportive, direct care providers were concerned that patient requests for assistance in using the technology would place increased demands on their time. Administrators and IT experts recognized issues such as privacy and costs related to offering these technologies throughout an organization and in the larger health care system.

**Conclusions:**

This study affirmed the acceptability of offering internet-based communication and information technologies on palliative care in-patient units. It provides the foundation for trialing these technologies on a palliative in-patient unit. Further study is needed to confirm the feasibility of offering these technologies at the bedside.

## Background

The quality of life in terminally ill patients is challenged by physical, emotional, social, and spiritual concerns [1]. Palliative care is important for patients who experience significant burden of illness at any stage along the disease trajectory; helping them and their families adapt to these challenges and improve their quality of life in existential, spiritual, psychological, and social domains, despite a decline in their overall health [2]. Along with managing physical symptoms, healthcare providers must try to ameliorate psychological suffering by attending to a broad range of psychosocial needs of patients and family members [3, 4]. Many factors contribute to psychological well-being and the quality of life in terminally ill patients, but social support and social connectedness play a key role. Maintaining relationships, open communication and connections with family and friends are ways of bolstering patient well-being [5].

Communication technologies make it possible for patients to be in touch with people who are important to them anytime, anywhere. Studies have reported the application of communication and information technologies in a variety of healthcare contexts and describe the benefits associated with the use of these technologies. For example, video-calling was tested with older adults and was shown to enhance quality of interactions between institutionalized elders and family [6] and helped relatives of cognitively impaired nursing home residents stay more involved in the caring process [7]. Home video was used to manage the care of chronically ill, high-risk patients and had positive effects on patient knowledge, social support, and clinical outcomes [8, 9]. The potential of videoconferencing and the internet to improve communication in hospice settings has also been identified [10]. Bender et al. [11] described an array of computer-mediated communication formats currently in use to support cancer patients and their families. The formats ranged from e-mail to chat rooms and social network sites; each offered peer-to-peer collaborative, interactive information sharing and support. In a study by Brecher [12], the use of Skype (a brand of voice-over-internet protocol) in a palliative care in-patient setting enhanced patient quality of life and connection to their loved ones. Skype has also been reported as being helpful in facilitating communication between care providers, patients and families [13]. While many healthcare institutions provide access to the internet for use by patients and families supplying their own equipment, the use and impact of the internet in these settings has not been systematically studied. Equally important is that most of these studies have focused on improving the clinical care of patients, not necessarily focused on the dimensions of social support and social connectedness.

People nearing the end of life need to keep in contact with their loved ones. However, regular social contact may be especially difficult for the terminally ill. For individuals in an in-patient palliative care ward, the social and psychological strain is amplified because of the isolating nature of being cared for in an institutional setting, away from family and friends. Technology cannot necessarily solve these problems, but it can mitigate some of the distress by facilitating social connection [14]. For communication and information technology to be successful, it is important to ascertain if patients and families consider it beneficial and desirable [15]. Hospice and palliative professionals also need to be open to such technology as a viable and user-friendly method that can be integrated into care [16]. This study was undertaken to consult a diverse group of key informants—including administrators, communication and information technology experts, direct care providers, patients and families—about their perspectives on introducing internet-based communication and information technologies on a palliative care in-patient unit; and to elicit feedback about perceptions of drawbacks and benefits such as enhancing social connectedness [14], facilitating communication with family members [12, 13], providing strong family support [12], and relaxation and entertainment [17].

## Methods

We conducted a 2 phase study entitled “Keep in Touch” (KIT), in order to explore the feasibility of introducing internet-based communication and information technologies in a specialized palliative care in-patient unit and to identify barriers and opportunities associated with using these technologies. In this first phase of the study, a diverse group of key informants were consulted regarding their perspectives on web-based communication on in-patient palliative care units. In phase 2 we conducted a feasibility study, to be reported separately. This study was conducted on a 30-bed specialty in-patient palliative care unit, located within a hospital in Winnipeg, Canada.

### Sample

A convenience sample of four sets of key informants, including health care facility administrators, communication and information technology (IT) experts, direct care providers, and patients receiving palliative care and their family members were recruited to participate in the study. Administrators were eligible if they had senior decision-making responsibilities and/or were in positions deemed relevant to the goals of the study, such as the privacy officer, manager of plant and facilities and the palliative care unit manager. IT experts were identified and selected, based on their direct or indirect association with the participating institution. IT experts were eligible if they had experience dealing with end-users of common information and communication technologies. These experts were either directly employed by the institution and had technology responsibilities; were technology service providers to the institution; or had technology retail or design backgrounds. Care providers were those who were employed on the palliative in-patient unit and provided direct care to patients. An invitation was sent to each eligible participant via e-mail and a promotional notice was posted in the unit conference room.

Patients were eligible if they were 18 years of age or older, able to speak and understand English; determined by their physicians to have a life-limiting illness with approximately 6 months or less to live; showed no evidence of confusion or delirium as identified by clinical consensus; were able to provide valid informed consent; and were receiving in-patient care on the participating palliative care unit. Family members of patients and bereaved family members were eligible if they were 18 years of age or older, able to speak and understand English, and if their family member was an in-patient on the unit anytime in the 12 months prior to commencing the study. Bereaved family members were identified by the unit manager; they were mailed letters of invitation asking their permission for research staff to make contact. For patients and family on the in-patient unit, promotional invitation posters were hung in five strategic locations approximately 1 week prior to the “drop-in” consultation sessions.

### Data collection

Data was collected over a course of several months between 2011 and 2012. Specific questions asked of each key informant group are shown in Table 1. Given that daily use of technologies may influence how people perceive and value information and communication technologies, all participants, with the exception of the IT participants, were asked about their own access to and use of the computer/internet. Consents and demographic information were collected prior to data collection. Two 2-hour focus groups facilitated by our research staff were conducted; one with institutional administrators and one with IT experts. We hosted two drop-in sessions in the large public dining room on the in-patient unit to consult direct care providers. These sessions enabled direct care providers working on the unit to provide input at their convenience. Questions were written out on flip chart paper and posted around the room; providers were asked to read and respond by posting their comments on colorful self-adhesive note paper on the flip chart paper. Two similar drop-in consultations were held on the unit with current patients and family members on the in-patient unit. Two patients and four family members on the unit attended. One family member was interviewed at a time more convenient for her on the unit. The two bereaved family members were interviewed together at a time and location convenient to them.

**Table 1:** Questions asked in each consultation group

Patients and Family Members
1. How do you keep in touch with your family and friends while you are here in hospital? 2. What effect does being in hospital have on people’s ability to stay in touch with family and friends? How does being in hospital affect patients’ ability to stay in touch with family and friends? How does being in hospital affect family members’ ability to stay in touch with other family and friends? 3. What advantages, if any, do you see to having internet access in patient care rooms on the ward? For patients? For patients’ family members or friends? 4. What drawbacks, if any, do you see to having internet access in patients’ rooms on the ward? For patients? For patients’ family members or friends? 5. What kinds of activities do you think people would use access to the internet to do, if it was available in patient care rooms? What would patients use it for? What would family use it for? 6. Would you be likely to use your own computer, tablet, or smart phone while on the ward or would you prefer to use a computer supplied by the hospital? 7. Would you be willing to pay to use a computer with internet access? If so, what would be a reasonable daily rate for a computer with unlimited internet access? 8. Other comments?
Direct Health Care Providers
1. Can you describe a situation in you think computer-mediated communication or information technology available at the patient’s bedside may have enhanced a patient’s quality of life during their hospital stay? Enhanced patient care? 2. What observations have you made regarding what patients or their family members are doing now to use computer-mediated communication technology at this Health Centre (e.g. using smart phones, using computers in public spaces)? 3. In what ways do you think access to computer-mediated communication and information technologies at the bedside might enhance patients’ quality of life during their hospital stay? Enhance patient care? Create opportunities for providing care? 4. How do you think patients might use computer-mediated communication or information technology at their bedside? 5. What are the characteristics of patients (or family members) you think are most likely to use computer-mediated communication or information technology at the patient’s bedside? 6. Might there be drawbacks to having computer-mediated communication or information technology available at a patient’s bedside? If so, what disadvantages do you see? Can you suggest ways that the drawbacks could be mitigated? 7. What concerns, if any, do you have about computer-mediated communication or information technology being available at the patient’s bedside?
Health Centre Administrators
1. In your opinion, would offering computer-mediated communication and information technologies to patients at the bedside fit the mission and values of this Health Centre? 2. What observations have you made regarding what patients or their family members are doing now to use computer-mediated communication technology at this Health Centre (e.g. using smart phones, using computers in public spaces)? 3. In what ways do you think access to computer-mediated communication and information technologies at the bedside might enhance patients’ quality of life during their hospital stay? Enhance patient care? Create opportunities for providing care? 4. Might there be drawbacks to having computer-mediated communication or information technology available at a patient’s bedside? If so, what disadvantages do you see? Can you suggest ways that the drawbacks could be mitigated? 5. From the perspective of the organization, what concerns, if any, do you have about computer-mediated communication or information technology being available at the patient’s bedside? 6. Again, from the perspective of the organization, what barriers do you foresee with implementing this service? How might these barriers be overcome? 7. Are you aware of any similar services offered to patients, either in Manitoba or elsewhere?
Communication and Information Technology Experts
1. What technology (that is, hardware, software, peripherals) do you recommend to achieve the stated functions and to address other factors such as privacy, ease of use, human-computer interface, and robustness? 2. What problems might arise from use of the technology by potentially frail and vulnerable patients, which may not be obvious to technologically-naïve health care providers? How might these problems be overcome? 3. Are there other communication and information functions that are not listed above, which could be helpful to patients and their families for keeping in touch with one another? What might be the IT-support demands associated with providing communication and information technologies to patients in hospital? 4. Given that technology changes rapidly, what is the expected lifespan of the recommended technology? How easily could it be upgraded? Can you suggest questions that we have not asked that we should ask?

### Data analysis

Descriptive statistics were used to analyze demographic data and quantitative data. Qualitative data was analyzed using content analysis, and constant comparative techniques to identify themes, consensus, and major differences arising from the perspectives of patients and family members, direct care providers, IT experts and administrators [18].

## Results

### Characteristics of participants

A total of 53 participants were recruited; 2 patients and 7 family members, 29 direct care providers, 8 IT experts, and 7 administrators. Table 2 illustrates the characteristics of direct care providers, IT experts, and administrator. Characteristics of patients and family members are shown in Table 3.

**Table 2:** Characteristics of direct care providers, IT experts and administrators (*N* = 44)

Participants characteristics	*N* (%)
Age	
≤ 30	4 (9.1)
31–40	11 (25.0)
41–50	8 (18.2)
51–60	15 (34.1)
≥ 61	4 (9.1)
Missing	2 (4.5)
Gender	
Female	25 (56.8)
Male	19 (43.2)
Education	
Completed post-secondary	36 (81.8)
Some post-secondary	7 (15.9)
High school	1 (2.3)
Occupation	
Nurse	12 (27.3)
HCA/clerical	8 (18.2)
Manager	7 (15.9)
Doctor	5 (11.4)
Other professional	5 (11.4)
Senior executive	4 (9.1)
Info technologist	3 (6.8)
Years in current occupation	
≤ 5	9 (20.5)
6–15	18 (40.9)
16–25	6 (13.6)
26–35	6 (13.6)
≥ 36	5 (11.4)

**Table 3:** Characteristics of patients and family members (*N* = 9)

Participants characteristics	*N* (%)
Age	
51–60	4 (44.4)
≥ 61	4 (44.4)
Missing	1 (11.1)
Gender	
Female	6 (66.7)
Male	3 (33.3)
Education	
Completed post-secondary	3 (33.3)
Some post-secondary	2 (22.2)
High school	3 (33.3)
Missing	1 (11.1)
Relationship to patient	
Patient	2 (22.2)
Spouse	3 (33.3)
Son or Daughter	3 (33.3)
Sibling	1 (11.1)
Marital status	
Married	5 (55.6)
Widowed	2 (22.2)
Divorced	1 (11.1)
Missing	1 (11.1)

### Participants’ experience with computers and internet

In terms of their own access to and use of the computer/internet, all but one of the respondents who used a computer also used the internet. Of 25 care providers who responded, 15 (51.8 %) used the computer/internet daily or several times a day, 5 (17.2 %) used the computer/internet nearly every day; 3 (10.3 %) used it at least once a week, and the remaining 2 (6.9 %) used the computer/internet less than once a month. Administrators used the computer/internet daily or several times a day. Of 9 family members or patients, 5 (55.6 %) use the computer/internet daily or several times a day. Three (33.3 %) used the computer/internet nearly every day and one person, at least once a month. Of the 40 respondents who reported using the internet, 29 (72.5 %) used it for e-mail or finding information and for at least one other purpose such as text messaging, social networking, or calling. Ten (25 %) reported using the internet for e-mail and finding information. One used the internet for finding information only.

### Estimates of patients and family use of computer/internet while hospitalized

Two patients and seven family members reported the frequency with which they used a computer/internet while in hospital and estimated the frequency with which they might possibly use a computer/internet if it was available in the hospital room. Although the number of participants was small, both patients and family members indicated they would frequently use this technology if available at the bedside.

### Current challenges of hospitalized patients’ ability to keep in touch

As patients admitted to the in-patient palliative care unit are typically quite ill, it is not surprising that they identified feeling too sick to call or text as a major challenge to staying in touch. As this participant noted, “[Hospitalization] hinders the ability to keep in touch depending on how you are feeling. Not always able to make phone calls. Sometimes rather e-mail friends and families.” [PR001].

Other factors were identified as making it challenging for patients and family members to keep in touch. Poor cellular phone reception in the hospital hampered the ability to stay connected. Cell phone use was described as being very expensive. For example, one patient stated that a pay-as-you-go cell phone account ran out of minutes rather quickly and that they used their cell phone only when necessary as it was too expensive to make long-distance calls. Having family who lived far away from the hospital made patient-family connections difficult either because of the distance to be travelled, problems associated with inclement weather, and difficulties securing transportation. Finally, the inability of family to visit because of their own health problems or lack of time also made it difficult for patients and families to keep in touch.

### Current and potential use of communication and information technology during hospitalization

#### The perception of patients and family members

While calling or text messaging on cell phones was identified by patients and families as a way of keeping in touch, they observed that in addition to poor reception, patients may quickly lose the ability to text due to functional decline. For example, one family member stated:

“She used her cell phone until she cannot text anymore. [There is a] phone in room but she cannot answer it by herself so someone has to be around to put it to her ear. Therefore, she is dependent on them to come.” [PF003]

Family members used the publicly accessible computers to send e-mails or access social networking internet sites. Patients and family members identified many examples of ways they would potentially use computers with internet access, were they available at the bedside. See Table 4 for details.

**Table 4:** Activities patients and family members suggested they might undertake if computers were at the bedside

Patients	Family members
• Entertainment such as playing games, movies, music, “looking at interesting things I find on the web” • Accessing information, medical or to satisfy curiosity • Contacting people via e-mail or “Skype visits”; “having e-mail messages read to her” • Looking at family photos • “He might have sent messages to the kids”	• Communication; e-mail • Entertainment such as playing games or other amusement such as taking part in surveys, looking for sales or store coupons, or “I looked up results for the Barrett Jackson Automobile auction.” • Keeping up with the latest news • Conducting personal business such as booking airline tickets, retrieving boarding passes, or on-line banking; allowing people to “follow up on stuff right away rather than wait to get home”

Given the choice between using their own computer or one provided by the hospital, seven out of eight respondents would prefer a computer supplied by the hospital. Five of seven participants were willing to pay for internet access in the hospital. A reasonable daily rate for unlimited internet access was suggested by participants and ranged from $1.00 to $5.00 per day. One person suggested that the cost should be kept “low” and another proposed that a flat rate be charged for the duration of the hospital stay so there would be no need for renewals.

#### The perspective of direct care providers

Direct care providers observed frequent cell or smart phone usage by patients and family members and reported that poor reception can have a disruptive effect on other patients as people move about the ward trying to find better reception. For example:

“Families sometimes stand outside of other patients’ rooms to use their phones for reception due to “hot spots”, then they disrupt those patients and the patients hear everything said on the phone.” [FG002]

Direct care providers reported that family members were the main users of public computers as patients are generally too ill to leave their rooms. Computers were used to play games, check e-mail, keep in touch with family or friends, make travel arrangements, watch movies, or listen to music.

Care providers envisioned some quality-of-life enhancing activities in which patients and families could partake. These included: i) communication or maintaining connections; ii) information-seeking; iii) personal business; and iv) entertainment activities (See Table 5). In terms of opportunities for care that access to information and communication technologies might provide, care providers saw the potential for including geographically-distant family members in decision-making meetings, displaying diagnostic images, providing virtual tours of hospices or personal care homes, and accessing medical websites.

**Table 5:** Four categories of quality-of-life enhancing activities identified by care providers

*Examples of communication or maintaining-connection activities included:* • Allowing patients to keep in touch with family and friends from out of town or who cannot come to the hospital to visit • “… able to stay connected to family and friends via email/Facebook/Skype. This may be very important in a situation where mobility is an issue or/and when family/friends cannot visit frequently” • Able to talk and see loved ones on the computer; “give them a warmer feeling than talking on the phone” • Viewing photos of family/grandkids, weddings
*Examples of potential information-seeking activities included:* • Looking up information related to “the disease process or medication”, health topics • Canadian Virtual Hospice • Keeping informed on “what’s going on in the world”, weather, current events
*Examples of potential use of technology to conduct personal business included:* • Making travel arrangements • Maintaining work relationships • Online banking • Paying bills
*Examples of potential entertainment-related activities included:* • Watching movies, listening to music, playing games, watching YouTube • Reading books, poetry • Surfing the internet • Video streams or daily blogs

In terms of who would be most likely to use communication and information technologies at the bedside, participants suggested that younger people, and people with at least some familiarity with computers, would be the most likely users of technology although several others suggested that age is no barrier to technology use and that learning to use a computer might be a welcome activity to help someone keep their mind off their illness.

#### The perspective of administrators

Administrators observed signs of growing public expectations regarding the availability of access to new modes of communication. By example, they cited the pervasive use of cell phones by patients and families throughout the facility, the use of publicly accessible computers on the care unit and in the hospital cafeteria; and requests for access to “Skype” via receipt of comment cards.

Administrators agreed that communication and information technologies would benefit patients’ quality of life. They also provided examples of applications of these technologies that have the potential to enhance patient care, such as using the technology for care decision-making conferences between team and geographically-distant family members, using the technology to bring diagnostic imaging results to the patient’s bedside, or conducting patient education or sharing information with the patient via the internet.

#### The perspective of IT experts

IT experts were provided with a list of desired functions of the information and communication technology (See Table 6). When asked if there were any additional functions that might be offered to patients or family members, participants suggested the opportunity to create legacy projects (such as using the timeline on Facebook or audio recordings). One participant described what he called a family “treasure box", which might include archived home movies, or poignant video, audio or text messages.

**Table 6:** A list of functions desired of the information and communication technology

*Functions Desired of the Communication and Information Technology:*	
• E-mail • Electronic chatting, texting • Access to social networking • Real-time audio and video communication for one-to-one conversations or for visits or meetings between groups of people (e.g. Skype) • Unlimited internet access • Access to on-line, timely, expert-mediated palliative care information and advice—i.e. Canadian Virtual Hospice (virtualhospice.ca) • Means for health care providers to conduct individual patient and family care planning conferences or to do patient/family teaching	

### Practical considerations of offering communication and information technology for palliative in-patients

Administrators stated that offering internet-based, computer-mediated communication and information technologies to patients at the bedside is consistent with “patient- and resident-centred” care and is a desirable goal. They addressed the importance of matching the technology to user capabilities and to the functions expected of the technology. Palliative patients are a challenging user population; most of them are in hospital a short time (18 days on average) although about a third of patients will have longer hospital stays. Physical and cognitive functions are likely to decline over time. It was observed that family members will be as likely to use information and communication technologies, and possibly more intensively, than patients.

Given observations about user characteristics and the limitations associated with specific types of hardware, it came as no surprise that the IT experts were reluctant to recommend specific hardware, software, or peripherals for use. However, they advised keeping the interface between users and whatever devices are chosen as simple and intuitive as possible, providing an integrated system that allows patients/families to perform a number of functions using one device, and making use of built-in accessibility features.

### Advantages and issues/concerns associated with communication and information technology

#### Patients and family

Patients and family listed a number of advantages to having internet access in patients’ rooms rather than in a public area on the ward; such as enhancing feeling of connectedness, reducing boredom, and more easily keeping in touch with loved ones. As aptly stated by this participant:

“It would allow them to communicate when it is convenient for the patient. The challenge is that I don’t always feel up to writing on the computer and when I do feel up to it, it’s more difficult to leave my room (e.g., get my gown and shoes on, etc.).” [PR002].

It also would allow family members to not have to leave the patient’s room, give them opportunities to catch up on work when the patient was resting, or save them time when they got home at the end of the day from sending emails updating family and friends.

“I find when I go home from here, I have several e-mails and phone calls to deal with; I run out of time. If I could make contact from here, it would be easier.” [PF001]

“I could do my e-mail. I could stay with my Mom but still do whatever I wanted to do on the internet. I could show my Mom things on the internet.” [PF005]

Several respondents said they could imagine no drawbacks to having the internet available in patients’ rooms while a few others worried that having computer access in the patient room might detract from the patient and the time spent visiting. See Table 7 for details.

**Table 7:** Perceived advantages and drawbacks of having internet access in patient care rooms

Patients and Family *Advantages:* • Not having to leave the patient’s room to use the computer. One patient said “The challenge is that I don’t always feel up to writing on the computer and when I do feel up to it, it’s more difficult to leave my room…have to put on my dressing gown and shoes…” • Feeling more connected to the world with easy access to family and friends without leaving the room • Reduced boredom • Able to communicate right away before losing concentration • Easier to deal with phone calls and email before going home • Staying by the patient’s bedside while keeping the rest of family and friends updated • Convenience • “I could show my Mom (patient) things on the internet”
*Drawbacks:* • Inappropriate use of the internet by family members or unsupervised children • Noise for the patient • Tying up staff time with questions about the computer • Family members focusing on the computer rather than on the patient
Health Care Providers *Advantages:* • Improved psychosocial wellness, less boredom, decreased feelings of isolation, decreased separation anxiety especially for parents with children • Feelings of support from family and friends • Distraction from illness • Boosting spirit, feeling better emotionally • Finding groups who have the same illness • “See places you have always wanted to see”
*Drawbacks:* • Patients accessing incorrect or misleading information related to their health • Noise for the patient • Family members paying more attention to the computer than to the patient or fewer in-person visits • Demands on staff time for trouble-shooting related to the technology • Equipment damage or loss • Accessing inappropriate websites • Additional “clutter” in the room

#### Direct care providers

Direct care providers suggested that offering information and communication technology at the bedside could enhance patients’ quality of life and particularly focused on how it would reduce patient boredom. Direct care providers saw this technology as allowing patients to watch movies, view pictures, access information, read newspapers and “to virtually see a loved one whether they be far or near without [the patient] having to leave their room and [family and friends their] home.” [FG001]. In terms of potential drawbacks or concerns the technology might bring about, while several participants indicated that they had no concerns or foresaw no drawbacks, others identified several potential issues, such as demands on staff time for trouble-shooting related to the technology and equipment damage or loss. See Table 7.

#### Administrators

Administrators foresaw that offering communication and information technology to patients and/or their families could enhance quality of life by allowing greater ease in communication and by simply being an extension of what patients and families are already doing at home. Identified issues related to offering these technologies throughout an organization and in the larger health care system included: financial implications, internet privacy, security and risk management, and human resource capability. See Table 8.

**Table 8:** Issues raised by administrators

Issues raised by administrators at an organizational level	
Financing	Insured vs. noninsured services Costs associated with implementation, material, service contracts, ongoing maintenance and upgrades
Human resources	System maintenance Front-line technology support Facilitating patient/resident use of technology
Privacy	Patients Healthcare providers
Security	System Equipment loss or damage Inappropriate internet access or activities

#### IT experts

Table 9 shows the issues raised by IT experts at the level of end users (patients, families, care providers) and at the level of health care organizations or systems. Two major issues with the most direct effect on patients, or their family members, concerned privacy and the need for human resources in order to be able to help patients effectively use the technology. Questions were raised about who assures data security and who assumes the risk for breaches of security and privacy while patients are in the care of health facilities. IT experts recognized that users need technical support. It was suggested that patients, especially, would likely benefit from assistance that not only solved technical problems when they arise but would also facilitate use of the technology so the patient would be able to imagine the technology’s potential and then achieve the most meaningful use of the technology during their hospital stay.

**Table 9:** Issues raised by IT experts

Individual level	Organization or system level
Privacy IT support Managing user expectations Managing service equality	Privacy and security Infrastructure capable of handling high demand Importance of planning and design Risk management

## Discussion

This study was designed to provide insights into the feasibility of offering communication and information technologies in a palliative care in-patient unit. A comprehensive understanding of issues associated with the use of these technologies from relevant parties is a critical factor for its successful implementation [19]. Therefore, we consulted people who would offer informed opinions on the value of, and issues associated with, offering internet-based information and communication technology at the bedside of palliative care patients.

Findings showed that most patients and family members would be more likely to use the internet-based communication and information technology if the technology was available at the bedside. However, three family members reported that their sick relative had no interest in using such technology even if it was available to them. Therefore, it is important to acknowledge that not all patients/families require or desire communication technology and that the application of such technology should be tailored to patients’ and family’s needs and preference. Potential use of this technology identified by participants reflected the ways in which they currently use their computers for communication, information-gathering, and entertainment.

Offering internet-based communication and information technology in the palliative care in-patient setting is challenging. It must be implemented such that the benefits outweigh the drawbacks. In this study, each participating group saw positive and imaginative applications of communication and information technology from their own unique vantage points. Patients and family members expressed their interest in having communication and information technology at their bedside and agreed that such technology would enhance their ability to keep in touch with family and friends. Care providers suggested that the proposed technology could enrich patients’ quality of life and care. As Mahon [20] advises, decisions about the use of technology must be made in the context of the goals of care. In this study, administrators appraised the application of communication and information technology at the organizational level and affirmed that offering such technology on the in-patient unit is consistent with the patient- and resident-centered mandate of the organization.

André argues that the patient’s health condition affects their perception of whether technology is a useful tool or not [19]. For those with terminal illness, loneliness can be particularly significant because declining health and hospitalization make close contact with loved ones more difficult [21, 22]. Palliative patients can mitigate loneliness by connecting to others [23]. Patients in this study indicated that internet-based communication and information technology might help them to connect with the world by overcoming barriers such as physical limitation, distance and transportation problems; this advantage was also identified by family members. This sentiment has been validated by other studies showing that internet-based communication technology such as Skype could comfort patients by allowing patients and family to be “together” online [12].

The conflict between “high tech” and “high touch” [15, 19] was also raised by participants in this study. Health care providers were concerned that offering communication and information technology to patients might result in more attention to the computer or fewer in-person visits. That said, technology should not replace in-person contact but rather should be used as a supplement [15]. In addition, Administrators and IT experts also raised issues of client and staff privacy and information and equipment security. These concerns have been commonly recognized in previous studies [12, 13].

## Conclusion

Patients, families, IT experts, and direct care providers in this study indicated that they saw many advantages, and very few disadvantages, to offering internet-based communication and information technologies at the bedside. Administrators acknowledged that offering these technologies is consistent with a patient-centred care philosophy, but they were also concerned about security and privacy issues. The findings of this first phase of the Keep in Touch study can help inform decisions related to the long-term sustainability of offering internet-based communication and information technologies within in-patient palliative care settings. Further study is needed to confirm the feasibility of offering internet-based communication and information technologies at the bedside.
